# Association between socioeconomic status and arteriosclerotic cardiovascular disease risk and cause-specific and all-cause mortality: Data from the 2005–2018 National Health and Nutrition Examination Survey

**DOI:** 10.3389/fpubh.2022.1017271

**Published:** 2022-11-22

**Authors:** Ruihuan Shen, Ning Zhao, Jia Wang, Peiyao Guo, Shuhui Shen, Detong Liu, Donghao Liu, Tong Zou

**Affiliations:** ^1^Department of Cardiology, National Center of Gerontology, Institute of Geriatric Medicine, Beijing Hospital, Chinese Academy of Medical Sciences, Peking Union Medical College, Beijing, China; ^2^Department of Gastrointestinal and General Surgery, National Center of Gerontology, Institute of Geriatric Medicine, Beijing Hospital, Chinese Academy of Medical Sciences, Peking Union Medical College, Beijing, China; ^3^Department of Cardiology, National Center of Gerontology, Institute of Geriatric Medicine, Beijing Hospital, Peking University Fifth School of Clinical Medicine, Beijing, China; ^4^Department of Oncology, National Center of Gerontology, Institute of Geriatric Medicine, Beijing Hospital, Chinese Academy of Medical Sciences, Peking Union Medical College, Beijing, China

**Keywords:** arteriosclerotic cardiovascular disease, socioeconomic, poverty income ratio, mortality, National Health and Nutrition Examination Survey (NHANES)

## Abstract

**Background:**

Morbidity and mortality of arteriosclerotic cardiovascular disease (ASCVD) varied according to socioeconomic status (SES), and evidence on the association between SES and ASCVD risk, and cause-specific and all-cause mortality was nevertheless lacking in large-scale or population-based studies.

**Methods:**

A multicycle cross-sectional design and mortality linkage study was conducted using data from Continuous National Health and Nutrition Examination Survey (NHANES) in the United States, including public use linked mortality follow-up files through December 31, 2019. Poverty income ratio (PIR) served as a SES index. A series of weighted Logistic regressions and Cox proportional hazards regressions were used to investigate the association between the SES and the risk of ASCVD and mortality, respectively.

**Results:**

The study sample was comprised of 30,040 participants aged 20–85 years old during the 2005–2018 period. Weighted Logistic regression models consistently indicated significant relationship between people experiencing poverty and increased risk of ASCVD, and linear trend tests were all statistically significant (all *P* for trend < 0.001). Additionally, weighted Cox regression analysis consistently demonstrated that the hazards of cause-specific and all-cause mortality increased, with the decrease of each additional income level, and trend analyses indicated similar results (all *P* for trend < 0.001).

**Conclusions:**

Our study confirmed that the SES was strongly linked to living with ASCVD, and cause-specific and all-cause mortality, even after adjusting for other factors that could impact risk, such as the American Heart Association (AHA)'s Life's Simple 7 cardiovascular health score and variables of age, sex, marital status, education, and depression severity.

## Introduction

At the global level, cardiovascular disease (CVD) was a significant public health issue, accounting for around 30% of the yearly global mortality rate (~17.6 million persons per year) and 10% of the global disease burden (1, 2). And at a country level, CVD morbidity and mortality have declined in several high-income nations, but they continued to rise significantly in low- and middle-income nations in recent years (3). CVD was the leading cause of death in the United States, accounting for almost one-third of all deaths (4). In 2009, it was estimated that the total direct and indirect expenses of CVD in the United States were $312.6 billion (5). At the individual level, behavioral, medicinal, and/or surgical treatments were frequently required in response to these symptoms associated with that condition, which can adversely affect one's quality of life.

Morbidity and mortality of CVD varied according to SES (6). Even though most nations and areas have experienced socioeconomic progress and greatly enhanced the people's material living standards over the past few decades, the bulk of the nation's massive wealth gap still exists (7). From 2010 to 2018, CVD mortality in the general U.S. population remained stable, while the total number of CVD deaths rose (8). The reason for this was likely to be the lack of improvement in health and nutrition among people experiencing poverty, who were more susceptible to ASCVD (9). As a consequence of this health inequality, there may be differences in individual characteristics such as behavior or genetics, and more contextual factors such as the social and political environment (“the causes of the causes”) as well as the interaction between them (10). Even though ASCVD risk was associated with a higher burden of traditional risk factors among individuals with low SES. However, risk factor profiles may not fully account for the observed differences, suggesting that low SES itself and other upstream characteristics may be independent risk factors for ASCVD (11).

Therefore, large-scale and population-based study was conducted to assess the association between SES and ASCVD risk and cause-specific and all-cause mortality, utilizing data from the NHANES.

## Materials and methods

### Database

The National Center for Health Statistics (NCHS) of the Centers for Disease Control and Prevention (CDC) launched numerous cycles of the United States cross-sectional Continuous NHANES from 2005 to 2018, providing public use linked mortality follow-up files through December 31, 2019 (12). In addition, the National Center for Health Statistics (NCHS) has connected many demographic surveys to death certificate information from the National Death Index (NDI) (13).

The NHANES utilized a complex, stratified, multistage, probability cluster design to create a nationally representative survey of the health and nutritional status of the non-institutionalized civilian population in the United States, with detailed information available in the NHANES survey methods and analytic guidelines (14). Additionally, data on the nutritional health and condition of non-institutionalized civilians in the US population were acquired *via* a series of home interviews, examinations, and laboratory measurements.

The public-use versions of the linked mortality follow-up files provided the mortality data for adult participants, which consisted of mortality follow-up data from the date of survey participation through December 31, 2019, after the files have been processed to minimize the likelihood of participant identification (13).

### Study design and population

This was a cross-sectional study. The Continuous NHANES was used to collect data from 2005 to 2018 in 2-year increments for the initial sample. Only participants with available demographic data, and who answered the self-reported question medical conditions questionnaire (MCQ)160C—“Ever told you had coronary heart disease (CHD)?,” MCQ160D—Ever told you had angina/angina pectoris, MCQ160E—“Ever told you had heart attack,” or MCQ160F—“Ever told you had a stroke?” on the medical conditions section were included. Responses marked as “missing,” “refused,” or “do not know” were regarded as missing in the original NHANES surveys. Participants who lacked information for one of the study covariates specified below were disqualified from the statistical analysis of data.

### Data collection and weight selection

Demographic data such as age, sex, marital status, educational level, and PIR were collected using the Sample Person and Family Demographics questionnaires. For adults 20 and older, questions about smoking status and certain comorbid conditions were asked, in the home, by trained interviewers using the Computer-Assisted Personal Interviewing (CAPI) system.

Trained health technicians and interviewers were arranged to deliver standardized body measurements [e.g., blood pressure, body mass index (BMI), et al.] and questionnaires [e.g., depression severity, et al.] to survey participants at the mobile examination center (MEC). Specifically, after resting quietly in a sitting position for 5 min and determining the maximum inflation level (MIL), three consecutive blood pressure readings are obtained. If a blood pressure measurement is interrupted or incomplete, a fourth attempt may be made. All blood pressure determinations (systolic and diastolic) are taken in the MEC. The body measures data were collected in the MEC, and BMI was calculated as weight in kilograms divided by height in meters squared, and then rounded to one decimal place. Depression was measured using the Patient Health Questionnaire (PHQ-9), a nine-item screening instrument that asks questions about the frequency of symptoms of depression over the past 2 weeks. A final follow-up question assesses the overall impairment of the depressive symptoms. Response categories “not at all,” “several days,” “more than half the days,” and “nearly every day” were given a score ranging from 0 to 3. A total score was calculated ranging from 0 to 27.

The NHANES 2005–2018 MEC exam data weights were used in all analyses to take stratification and clustering into account because of the complex sample design.

### Primary study variables

#### Assessment of ASCVD

“Has a doctor or other health professional ever told {you/SP} that {you/s/he}... had a coronary heart disease/angina, also called angina pectoris/heart attack (also called myocardial infarction)/stroke?” was a question on the medical conditions section of the household questionnaires *via* home interview, and those who answered “yes” were deemed to have a history of ASCVD.

### Independent variable

#### Assessment of SES

PIR—an index for the ratio of family income to poverty served as a SES index in our study. The Department of Health and Human Services' (HHS) poverty guidelines were used as the poverty measure to calculate this index. These guidelines are issued each year, in the Federal Register, for determining financial eligibility for certain federal programs such as Head Start, Supplemental Nutrition Assistance Program (SNAP) (formerly Food Stamp Program), Special Supplemental Nutrition Program for Women, Infants, and Children (WIC), and the National School Lunch Program.

PIR was calculated by dividing family income by the poverty guidelines, specific to family size, as well as the appropriate year and state. The values were not computed if the income screener information [Income questionnaire (INQ) 220: <$20,000 or ≥$20,000] was the only family income information reported. If family income was reported as a range value, the midpoint of the range was used to compute the variable. The values of PIR at or above 5.00 were coded as 5.00 or more because of disclosure concerns. The values were not computed if the family income data was missing.

There were three distinct categories of *SES*: low income (PIR < 1.3), middle income (PIR = 1.3–3.5), and high income (PIR ≥ 3.5) (15, 16).

### Covariates and confounders

It was necessary to account for a number of possible confounding factors. Age and Life's Simple 7 cardiovascular health score were included in the analysis as continuous variables. The “Life's Simple 7” criteria, devised by the AHA to describe ideal cardiovascular health, included not smoking, regular physical activity, healthy diet, keeping normal weight, and controlling cholesterol, blood pressure, and blood glucose levels. The Life's Simple 7 cardiovascular health score varied from 0 to 14 (0 was the worst score and 14 was the optimal score) and was calculated by adding the number of ideal health metrics achieved. The sex was categorized as male and female. The marital status category included married, living with a partner, separated, divorced, widowed, and never married. The educational background was specified as college graduate or above, some college or AA degree, high school graduate, 9–11th grade, <9th grade. The categories for smoking status were former, current, and never. BMI was classified as low (i.e., <18.5), normal (i.e., 18.5–25), overweight (i.e., ≥25) (17). The PHQ-9 was used to determine the severity of depression; scores of 5, 10, 15, and 20 were used as the thresholds for mild, moderate, moderately severe, and severe depression, respectively (18, 19).

Accumulating evidence has shown that there were several disparities in risk factors and ASCVD among the general populations, including discrepancy in age (20, 21), sex (22–24), marital status (25, 26), educational level (27), depression severity (28), and the American Heart Association's Life's Simple 7 cardiovascular health score (29, 30).

### Comorbid conditions

Information on comorbidities was self-reported by participants. Regarding the question “Have you ever been told by a doctor or health professional that you have …?” persons who answered “yes” were perceived as having the following comorbidities: congestive heart failure (CHF) and arthritis.

Hypertension was diagnosed by: blood pressure/cholesterol (BPQ) 020: ever told you had high blood pressure; BPQ 030: told had high blood pressure−2+ times; BPQ 040a: taking prescription for hypertension; using anti-hypertension drug; judging hypertension on average blood pressure. And average blood pressure was calculated by the following protocol: 1. If only one blood pressure reading was obtained, that reading is the average. 2. If there is more than one blood pressure reading, the first reading is always excluded from the average. 3. If only two blood pressure readings were obtained, the second blood pressure reading is the average. 4. If all diastolic readings were zero, then the average would be zero.

Chronic obstructive pulmonary disease (COPD) was characterized by a reduction in expiratory air flow rates. This is defined in spirometry as a reduction in the ratio of the expiratory volume measured in the 1st second of a forceful exhalation (FEV_1_) to the total volume of air exhaled in a complete forced expiration (FVC), the FVC being an estimate of the individual's effective lung volume. Eligible participants performed an initial or “baseline” 1st test spirometry examination. Then a selected subsample of participants whose Baseline 1st Test Spirometry results showed a FEV_1_/FVC ratio below the lower limit of normal and/or below 70% were asked to repeat spirometry after inhaling a β_2_-adrenergic bronchodilator medication to open up their airways. This helps differentiate asthma from COPD (31). Asthma patients usually show improvements in post-bronchodilator spirometry testing, while patients with COPD exhibit little, if any, response to the medication. Spirometric testing using β_2_-adrenergic bronchodilator is routinely employed by clinicians to diagnose asthma in both children and adults, and current clinical practice guidelines (32) consider post-bronchodilator spirometry testing is essential for the initial diagnosis of asthma. Thus, COPD was diagnosed by: FEV_1_/FVC < 0.7 post-bronchodilator; MCQ160p: ever told you had emphysema; using drug: selective phosphodiesterase-4 inhibitors, mast cell stabilizers, leukotriene modifiers, inhaled corticosteroids, age above 40, with smoke history, or chronic bronchitis.

Other than that, Parkinson's disease was diagnosed by taking anti-Parkinson agents; and the diagnostic criteria for diabetes are: doctor told you to have diabetes; glycohemoglobin HbA1c (%) > 6.5; random blood glucose (mmol/L) ≥11.1; 2-h oral glucose tolerance test (OGTT) blood glucose (mmol/L) ≥11.1; use of diabetes medication or insulin.

### Follow-up and outcomes

The period of follow-up lasted from the date of the interview through the last follow-up time, December 31, 2019, or the date of death, whichever came first. Records from the NDI provided information on these included participants' causes of death. The endpoints for this study were all-cause mortality, which encompassed all known and unknown causes; cardiovascular mortality, which encompassed causes of diseases of heart and cerebrovascular diseases death.

### Statistical analysis

For categorical data (i.e., Sex, Marital Status, Educational level, Smoking Status, Depression Severity, BMI, CHF, CHD, Hypertension, ASCVD, Angina, Heart Attack, Stroke, Arthritis, COPD, Diabetes, PD), we used weighted proportions and corresponding 95% confidence intervals (CIs), while for continuous data (i.e., Age, PIR, the American Heart Association's Life's Simple 7 cardiovascular health score), we used weighted means and associated standard deviations (SDs). Design-based χ^2^-tests and Analysis of variance (ANOVA) were used to investigate whether categorical and continuous variables were associated with SES, respectively. The aforementioned χ^2^-test and ANOVA were survey-weighted models.

The weighted Kaplan–Meier curves were used to present the rate of cause-specific and all-cause mortality. Survival rates by SES were compared using the Mantel-Cox Log-rank test. The survival probabilities were estimated as the time intervals from the date of interview to the last follow-up time, December 31, 2019, or the date of death.

In order to find the association between SES and ASCVD risk and cause-specific and all-cause mortality, we conducted a sensitivity analysis by gradually adjusting for potential confounders. Specific details are described below: a series of weighted Logistic regressions analyses were conducted to assess the association of ASCVD risk with SES in various models following adjusting for potential confounders. Gradually, covariates were placed into the multivariable model by a priori selection. Crude and adjusted odds ratios (OR) and their 95% CIs between ASCVD risk and SES were reported. Similarly, a series of weighted Cox regressions analyses were conducted to estimate the associations between SES and the probabilities of cause-specific and all-cause death, after controlling for possible confounding factors in various models. The correlation between the SES and outcomes was provided as a crude and adjusted hazard ratio (HR) and its 95% CIs.

Our study defined the “high income,” which corresponded with the PIR ≥ 3.5, as the reference group. Trend analyses were conducted by entering the SES as continuous variable and rerunning the corresponding regression models.

NHANES (2012) has reported the warning on the analysis combing data across 2005–2006 and 2007 later due to survey design changes (33). Thus, sensitivity analysis on the estimates was performed. For the 2005–2006 and 2007–2018 survey periods, weighted Logistic regressions analyses were conducted to assess the association of ASCVD risk with SES in full adjusted models, respectively.

For statistical analysis, R (version 4.1.2; https://www.R-project.org) was utilized. Sampling design elements include the primary sampling units (PSUs), strata, and weights. However, ignoring the design elements that are included can often lead to inaccurate point estimates and/or inaccurate standard errors. Thus, the complexity of the sampling design was taken into account in each analysis by specifying PSUs, strata, and weights using the R package “survey” (version 4.1-1). We used MEC exam weights for all sample estimations (34–36). A two-sided *P* < 0.05 was considered statistically significant for testing the hypotheses of the study.

## Results

### Participant characteristics

According to inclusion and exclusion criteria, the unweighted sample for the final analysis consisted of 30,040 participants aged 20–85 from 2005 to 2018, representing 185.16 million non-institutionalized United States residents. The number of participants in the low income (weighted prevalence = 20.60%, 95% CI = 19.44–21.76%), middle income (weighted prevalence = 35.72%, 95% CI = 33.79–37.66%), and high income (weighted prevalence = 43.68%, 95% CI = 40.70–46.66%) levels were 9,232, 11,419, 9,389, respectively. while 2,929 participants (weighted prevalence = 7.68%, 95% CI = 7.14–8.22%) had ASCVD. These corresponded to 38.14, 66.14, 80.88, and 14.22 million adults in the general population, respectively. Characteristics of the study participants according to SES were presented in Table 1. Statistically significant differences were found for SES regarding socio-demographic, the physical and mental health-related factors, and comorbid conditions. Of note, Participants with lower incomes were characterized by younger, female, lower Life's Simple 7 cardiovascular health scores, low BMI, and current smokers, as well as higher rates of mild to severe depressive symptoms.

**Table 1:** Baseline characteristics of study participants^a^.

**Characteristic**	**Total**	**High income**	**Middle income**	**Low income**	***P*-value**
Age	47.37 ± 0.24	48.57 ± 0.30	47.97 ± 0.32	43.79 ± 0.46	<0.0001
Poverty income ratio	3.04 ± 0.03	4.69 ± 0.01	2.32 ± 0.01	0.79 ± 0.01	<0.0001
Life's simple 7	8.23 ± 0.03	8.61 ± 0.04	8.01 ± 0.04	7.80 ± 0.06	<0.0001
Sex (%)					<0.0001
Male	49.26 (47.10, 51.42)	51.44 (50.48, 52.39)	48.57 (47.58, 49.56)	45.83 (44.70, 46.96)	
Female	50.74 (48.47, 53.01)	48.56 (47.61, 49.52)	51.43 (50.44, 52.42)	54.17 (53.04, 55.30)	
Marital status (%)					<0.0001
Married	55.62 (52.35, 58.89)	68.36 (66.80, 69.92)	52.37 (50.54, 54.20)	34.23 (32.32, 36.15)	
Living with partner	8.16 (7.54, 8.79)	5.73 (5.00, 6.46)	8.33 (7.56, 9.10)	13.04 (11.84, 14.25)	
Separated	2.36 (2.13, 2.59)	1.10 (0.84, 1.36)	2.38 (2.02, 2.73)	5.02 (4.39, 5.65)	
Divorced	10.58 (9.90, 11.27)	8.14 (7.47, 8.81)	11.82 (10.97, 12.66)	13.62 (12.44, 14.80)	
Widowed	5.50 (5.10, 5.89)	2.99 (2.58, 3.39)	7.19 (6.58, 7.80)	7.88 (7.15, 8.60)	
Never married	17.78 (16.79, 18.77)	13.69 (12.56, 14.82)	17.92 (16.59, 19.25)	26.21 (23.67, 28.75)	
Educational level (%)					<0.0001
College graduate or above	30.02 (27.65, 32.40)	49.44 (47.07, 51.82)	18.51 (17.00, 20.03)	8.80 (7.58, 10.02)	
Some college or AA degree	31.82 (30.28, 33.36)	30.08 (28.49, 31.67)	35.66 (34.29, 37.03)	28.84 (26.75, 30.93)	
High school graduate	23.37 (21.86, 24.88)	16.01 (14.75, 17.27)	29.12 (27.66, 30.58)	29.00 (27.35, 30.65)	
9–11th grade	10.12 (9.25, 11.00)	3.77 (3.10, 4.44)	11.60 (10.59, 12.61)	21.05 (19.48, 22.62)	
< 9th grade	4.67 (4.22, 5.11)	0.70 (0.54, 0.87)	5.10 (4.47, 5.73)	12.31 (11.16, 13.45)	
Smoking status (%)					<0.0001
Never	54.58 (52.20, 56.95)	60.12 (58.60, 61.63)	52.11 (50.47, 53.74)	47.12 (45.10, 49.15)	
Former	25.09 (23.43, 26.74)	27.16 (25.77, 28.56)	26.08 (24.79, 27.37)	18.96 (17.83, 20.09)	
Current	20.33 (19.14, 21.53)	12.72 (11.69, 13.75)	21.81 (20.57, 23.05)	33.92 (31.85, 35.98)	
Depression severity (%)					<0.0001
None	77.20 (73.63, 80.76)	84.53 (83.62, 85.44)	75.26 (74.13, 76.39)	65.02 (63.55, 66.49)	
Mild	15.17 (14.43, 15.91)	11.52 (10.70, 12.34)	17.01 (16.06, 17.96)	19.71 (18.65, 20.77)	
Moderate	4.85 (4.45, 5.24)	2.79 (2.39, 3.20)	4.87 (4.36, 5.38)	9.15 (8.42, 9.89)	
Moderately severe	2.00 (1.78, 2.23)	0.85 (0.64, 1.05)	2.05 (1.70, 2.41)	4.37 (3.79, 4.94)	
Severe	0.78 (0.68, 0.89)	0.31 (0.18, 0.44)	0.81 (0.61, 1.01)	1.75 (1.41, 2.09)	
Body mass index (%)					<0.0001
Normal	28.10 (26.59, 29.61)	29.49 (28.03, 30.95)	26.58 (25.25, 27.90)	27.81 (26.28, 29.35)	
Overweight	70.38 (67.13, 73.63)	69.53 (67.99, 71.07)	71.78 (70.50, 73.07)	69.75 (68.07, 71.43)	
Low	1.51 (1.33, 1.70)	0.98 (0.71, 1.25)	1.64 (1.32, 1.95)	2.44 (1.98, 2.90)	
Comorbidity CHF (%)	2.23 (2.00, 2.46)	1.09 (0.87, 1.32)	2.96 (2.53, 3.39)	3.39 (2.91, 3.88)	<0.0001
Comorbidity CHD (%)	3.32 (2.95, 3.68)	2.86 (2.44, 3.28)	3.99 (3.47, 4.51)	3.12 (2.69, 3.54)	<0.001
Comorbidity hypertension (%)	38.08 (36.15, 40.01)	36.29 (34.70, 37.89)	40.04 (38.74, 41.34)	38.47 (36.81, 40.14)	<0.001
Comorbidity ASCVD (%)	7.68 (7.14, 8.22)	5.44 (4.94, 5.94)	9.13 (8.33, 9.92)	9.93 (9.17, 10.69)	<0.0001
Comorbidity angina (%)	2.14 (1.89, 2.39)	1.50 (1.19, 1.81)	2.59 (2.23, 2.96)	2.71 (2.33, 3.09)	<0.0001
Comorbidity heart attack (%)	3.26 (2.94, 3.58)	2.41 (2.01, 2.80)	3.59 (3.17, 4.01)	4.48 (3.95, 5.02)	<0.0001
Comorbidity stroke (%)	2.84 (2.60, 3.08)	1.55 (1.31, 1.80)	3.55 (3.09, 4.01)	4.34 (3.87, 4.81)	<0.0001
Comorbidity arthritis (%)	25.99 (24.44, 27.55)	24.82 (23.50, 26.15)	27.10 (25.76, 28.45)	26.55 (24.85, 28.26)	0.02
Comorbidity COPD (%)	4.55 (4.10, 5.01)	3.91 (3.37, 4.45)	4.72 (4.20, 5.25)	5.61 (4.83, 6.39)	<0.001
Comorbidity diabetes (%)					<0.0001
No	81.69 (78.06, 85.33)	84.29 (83.25, 85.33)	80.02 (78.87, 81.17)	79.10 (78.14, 80.05)	
Diabetes	13.37 (12.59, 14.14)	10.92 (10.03, 11.82)	14.91 (13.88, 15.94)	15.87 (14.92, 16.81)	
IGT	4.94 (4.54, 5.34)	4.79 (4.27, 5.31)	5.07 (4.48, 5.65)	5.03 (4.44, 5.63)	
Comorbidity PD (%)	0.94 (0.78, 1.10)	0.67 (0.46, 0.87)	1.10 (0.84, 1.36)	1.23 (0.93, 1.52)	0.003

a Data are expressed as weighted proportions and corresponding 95% confidence intervals for categorical variables and as weighted means and associated standard deviations (SDs) for continuous variables. Two-sided *P*s-values show results of univariate comparisons between different socioeconomic status. All categorical variables were tested with the χ^2^-test. Continuous variables were tested with Analysis of variance (ANOVA). The aforementioned χ^2^-test and ANOVA were survey-weighted models.

CHF, congestive heart failure; CHD, coronary heart disease; ASCVD, arteriosclerotic cardiovascular disease; COPD, chronic obstructive pulmonary disease; IGT, impaired glucose tolerance; PD, Parkinson's disease.

### The association of SES with ASCVD risk

Results of weighted Logistic regressions analyses of SES in relation to the risk of the ASCVD were displayed in Table 2. There were significant association between SES and increased risk of the ASCVD in model 1 (unadjusted model), model 2 [adjusted for age (continuous), sex (male or female), and marital status (Married, Living with partner, Separated, Divorced, Widowed, Never married), educational level (divided into <9th grade, 9–11th grade, high school graduate, some college or AA degree, college graduate or above), depression severity (None, Mild, Moderate, Moderately Severe, Severe)], and model 3 [further adjusted for the American Heart Association's Life's Simple 7 (continuous)]. For example, the result in model 3 showed that participants with the middle and low income, the risks of having ASCVD increased by 31% (OR = 1.31, 95% CI = 1.13–1.51), 80% (OR = 1.80, 95% CI = 1.55–2.10), respectively, compared with participants in the high-income level. And there was statistical significance in all of the trend analyses (all *P* for trend < 0.001).

**Table 2:** Crude and adjusted association socioeconomic status and increased arteriosclerotic cardiovascular disease risk.

**Model**	**Socioeconomic status (SES)**			***P*-value for trend**
	**High income (%) = 20.60**	**Middle income (%) = 35.72**	**Low income (%) = 43.68**	
	**(19.44, 21.76)**	**(33.79, 37.66)**	**(40.70, 46.66)**	
Model 1 (OR)	1.00 (Reference)	1.75 (1.53–2.00)	1.92 (1.69–2.17)	<0.001
*P*-values		<0.001	<0.001	
Model 2 (OR)	1.00 (Reference)	1.34 (1.16–1.55)	1.92 (1.65–2.23)	<0.001
*P*-values		<0.001	<0.001	
Model 3 (OR)	1.00 (Reference)	1.31 (1.13–1.51)	1.80 (1.55–2.10)	<0.001
*P*-values		<0.001	<0.001	

Model 1: Unadjusted model; Model 2: Adjusted for age (continuous), sex (male or female), and marital status (Married, Living with partner, Separated, Divorced, Widowed, Never married), educational level (divided into <9th grade, 9–11th grade, high school graduate, some college or AA degree, college graduate or above), depression severity (None, Mild, Moderate, Moderately Severe, Severe); Model 3: Further adjusted for the American Heart Association's Life's Simple 7 (continuous).

OR, odds ratio.

### Survival analysis

The leading causes of death in different SES were listed in Table 3. All-cause, cardiovascular, or malignant neoplasm mortality rates were 4.49, 1.20, and 1.36% for participants with high income levels, respectively; among middle-income leveled participants, mortality rates for all-causes, cardiovascular diseases, or malignant neoplasms were 9.34, 2.81, and 2.26%, respectively; and the prevalence of all-cause, cardiovascular, or malignant neoplasms mortality was 10.19, 2.85, and, 2.24% for low-income participants, respectively;

**Table 3:** The weighted prevalence of leading causes of death in different socioeconomic status.

**Cause of**	**High**	**Middle**	**Low**
**death**	**income**	**income**	**income**
Diseases of heart (%)	1.00	2.33	2.41
Cerebrovascular diseases (%)	0.20	0.48	0.44
Influenza and pneumonia (%)	0.08	0.12	0.15
Chronic lower respiratory diseases (%)	0.22	0.50	0.69
Nephritis, nephrotic syndrome and nephrosis (%)	0.02	0.18	0.23
Diabetes mellitus (%)	0.17	0.29	0.44
Malignant neoplasms (%)	1.36	2.26	2.24
Alzheimer's disease (%)	0.10	0.30	0.15
Accidents (unintentional injuries) (%)	0.19	0.33	0.36
All other causes (residual) (%)	1.15	2.55	3.08
All-cause (%)	4.49	9.34	10.19

**Figure 1:**
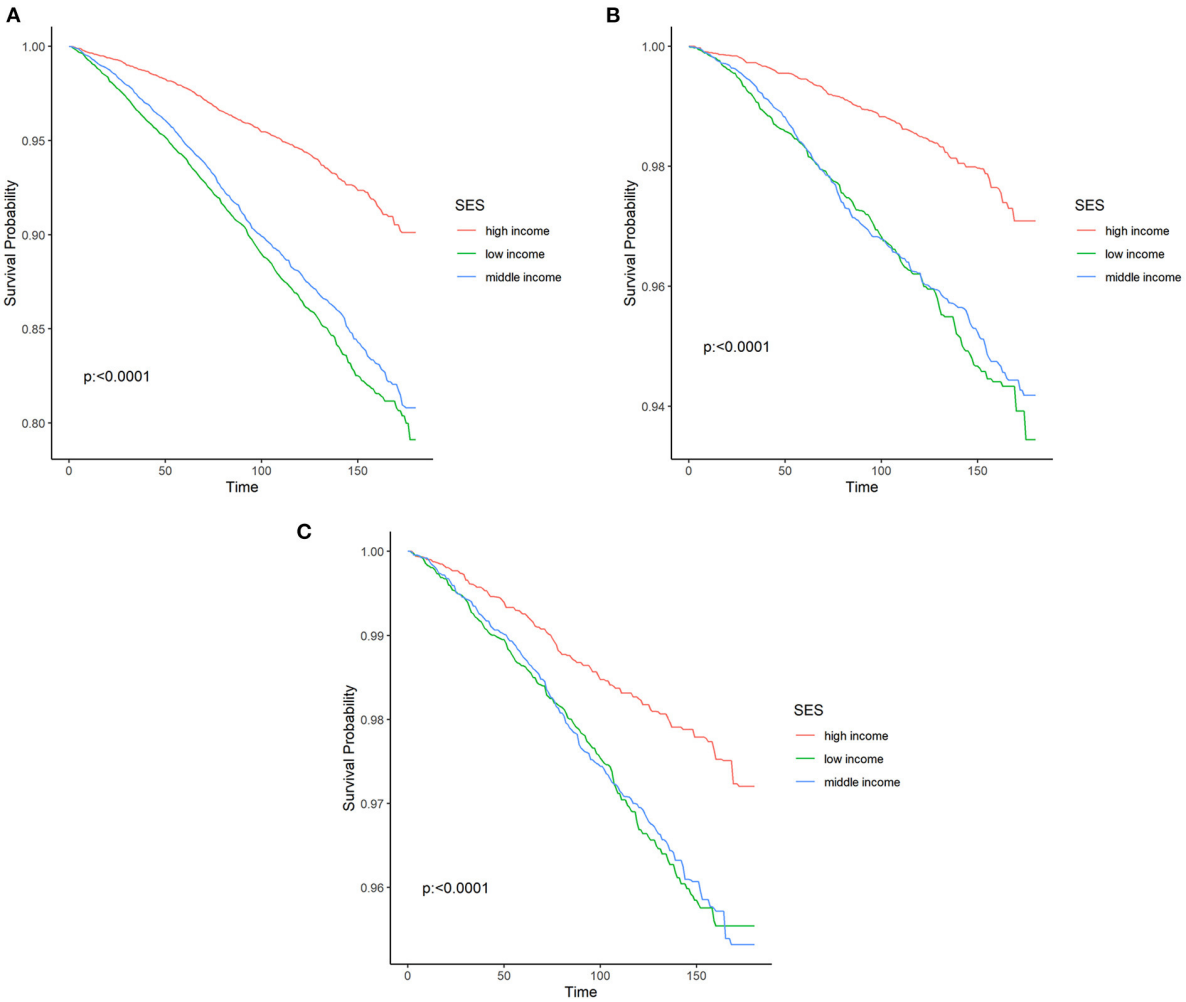
Kaplan–Meier curves were depicted to show the association of the socioeconomic status with all-cause **(A)**, cardiovascular **(B)**, malignant neoplasms **(C)** mortality, with follow-up in months. SES, socioeconomic status.

The Kaplan-Meier curves for all-cause, cardiovascular, and malignant neoplasms mortality were presented in Figures 1A–C, respectively. The median follow-up time from the date of interview to the last follow-up time, December 31, 2019, or the date of death was 89.00 months (ranged from 1 to 180 months). Moreover, the median age of participants at the date of interview was 49 years old (ranged from 20 to 85 years old), and the median age of participants at the last follow-up time, December 31, 2019, or the date of death was 57 years old (ranged from 21 to 99 years old). The upper red survival curve for high-income participants were all above the lower curve including blue survival curve for middle-income participants and green survival curve for low-income participants across the entire 180 months of follow-up (all log rank *P* < 0.001), visually indicating that survival probability of high incomes was greater than both middle- and low-income groups, suggesting a survival benefit.

The results from a series of weighted Cox regressions analyses in Table 4 consistently indicated that low-income participants were at a higher risk of all-cause death. For instance, A weighted multivariable Cox regression model 3 showed that after controlling for covariates, the hazards of all-cause death increased by 43% (HR = 1.43, 95% CI = 1.25–1.63) in middle income levels and 84% (HR = 1.84, 95% CI = 1.58–2.15) in low-income levels, compared to high income group. And trend analyses were all statistically significant (all *P* for trend < 0.001).

**Table 4:** Crude and adjusted association between socioeconomic status and all-cause mortality.

**Model**	**Socioeconomic status (SES)**			***P*-value for trend**
	**High income (%) = 20.60**	**Middle income (%) = 35.72**	**Low income (%) = 43.68**	
	**(19.44, 21.76)**	**(33.79, 37.66)**	**(40.70, 46.66)**	
Model 1 (HR)	1.00 (Reference)	2.19 (1.92–2.49)	2.46 (2.15–2.82)	<0.001
*P*-values		<0.001	<0.001	
Model 2 (HR)	1.00 (Reference)	1.46 (1.28–1.66)	1.93 (1.66–2.25)	<0.001
*P*-values		<0.001	<0.001	
Model 3 (HR)	1.00 (Reference)	1.43 (1.25–1.63)	1.84 (1.58–2.15)	<0.001
*P*-values		<0.001	<0.001	

Model 1: Unadjusted model; Model 2: Adjusted for age (continuous), sex (male or female), and marital status (Married, Living with partner, Separated, Divorced, Widowed, Never married), educational level (divided into <9th grade, 9–11th grade, high school graduate, some college or AA degree, college graduate or above), depression severity (None, Mild, Moderate, Moderately Severe, Severe); Model 3: Further adjusted for the American Heart Association's Life's Simple 7 (continuous).

HR, hazard ratio.

Similarly, a series of weighted Cox regressions analyses result in Table 5 consistently suggest that the hazards of cardiovascular mortality increased, with the decrease of each additional income level. Taking the weighted multivariable Cox regressions model 3 as an example, compared to high income group, the risks of cardiovascular death were increased by 43% (HR = 1.43, 95% CI = 1.13–1.81), and 81% (HR = 1.81, 95% CI = 1.41–2.33) for middle- and low-income participants, respectively, after multivariable adjustment. And statistical significance was found in all trend analyses (all *P* for trend < 0.001).

**Table 5:** Crude and adjusted association between socioeconomic status and cardiovascular disease mortality.

**Model**	**Socioeconomic status (SES)**			***P*-value for trend**
	**High income (%) = 20.60**	**Middle income (%) = 35.72**	**Low income (%) = 43.68**	
	**(19.44, 21.76)**	**(33.79, 37.66)**	**(40.70, 46.66)**	
Model 1 (HR)	1.00 (Reference)	2.45 (1.94–3.10)	2.58 (2.02–3.28)	<0.001
*P*-values		<0.001	<0.001	
Model 2 (HR)	1.00 (Reference)	1.47 (1.16–1.87)	1.90 (1.48–2.45)	<0.001
*P*-values		0.002	<0.001	
Model 3 (HR)	1.00 (Reference)	1.43 (1.13–1.81)	1.81 (1.41–2.33)	<0.001
*P*-values		0.003	<0.001	

Model 1: Unadjusted model; Model 2: Adjusted for age (continuous), sex (male or female), and marital status (Married, Living with partner, Separated, Divorced, Widowed, Never married), educational level (divided into <9th grade, 9–11th grade, high school graduate, some college or AA degree, college graduate or above), depression severity (None, Mild, Moderate, Moderately Severe, Severe); Model 3: Further adjusted for the American Heart Association's Life's Simple 7 (continuous).

HR, hazard ratio.

Likewise, the weighted Cox regressions analyses results were shown in Table 6, estimating the associations between the SES and the hazards of having malignant neoplasms mortality. A series of multivariable adjusted weighted Cox regressions consistently revealed that SES may contribute much to the risks of malignant neoplasms mortality. As a result of model 3, participants with middle and low income had increased hazards of malignant neoplasms mortality by 27% (HR = 1.27, 95% CI = 1.01–1.63) and 58% (HR = 1.58, 95% CI = 1.17–2.13), respectively, compared to participants with high income. And the trend analyses all showed statistical significance (all *P* for trend < 0.05).

**Table 6:** Crude and adjusted association between socioeconomic status and malignant neoplasms mortality.

**Model**	**Socioeconomic status (SES)**			***P*-value for trend**
	**High income (%) = 20.60**	**Middle income (%) = 35.72**	**Low income (%) = 43.68**	
	**(19.44, 21.76)**	**(33.79, 37.66)**	**(40.70, 46.66)**	
Model 1 (HR)	1.00 (Reference)	1.74 (1.36–2.23)	1.77 (1.37–2.29)	<0.001
*P*-values		<0.001	<0.001	
Model 2 (HR)	1.00 (Reference)	1.29 (1.01–1.65)	1.63 (1.22–2.18)	0.001
*P*-values		0.042	0.001	
Model 3 (HR)	1.00 (Reference)	1.27 (1.01–1.63)	1.58 (1.17–2.13)	0.003
*P*-values		0.045	0.003	

Model 1: Unadjusted model; Model 2: Adjusted for age (continuous), sex (male or female), and marital status (Married, Living with partner, Separated, Divorced, Widowed, Never married), educational level (divided into <9th grade, 9–11th grade, high school graduate, some college or AA degree, college graduate or above), depression severity (None, Mild, Moderate, Moderately Severe, Severe); Model 3: Further adjusted for the American Heart Association's Life's Simple 7 (continuous).

HR, hazard ratio.

### Sensitivity analysis

Results of weighted Logistic regressions analyses of SES in relation to the risk of the ASCVD for the 2005–2006 and 2007–2018 survey periods were presented in Table 7. The result showed that participants with the middle and low income, the risks of having ASCVD increased by 42% (OR = 1.42, 95% CI = 1.10–1.83), 103% (OR = 2.03, 95% CI = 1.22–3.36) in NHANES 2005–2006, and 29% (OR = 1.29, 95% CI = 1.10–1.51), 77% (OR = 1.77, 95% CI = 1.51–2.07) in NHANES 2007–2018, respectively, compared with participants in the high-income level after full adjustment. And there was statistical significance in all of the trend analyses (all *P* for trend < 0.05).

**Table 7:** Full Adjusted association socioeconomic status and increased arteriosclerotic cardiovascular disease risk for the 2005–2006 and 2007–2018 survey periods.

**Model**	**Socioeconomic status (SES)**			***P*-value for trend**
	**High income**	**Middle income**	**Low income**	
NHANES 2005–2006 (OR)	1.00 (Reference)	1.42 (1.10–1.83)	2.03 (1.22–3.36)	0.010
*P*-values		0.012	0.009	
NHANES 2007–2018 (OR)	1.00 (Reference)	1.29 (1.10–1.51)	1.77 (1.51–2.07)	<0.001
*P*-values		0.002	<0.001	

Models were adjusted for age (continuous), sex (male or female), and marital status (Married, Living with partner, Separated, Divorced, Widowed, Never married), educational level (divided into <9th grade, 9–11th grade, high school graduate, some college or AA degree, college graduate or above), depression severity (None, Mild, Moderate, Moderately Severe, Severe), and the American Heart Association's Life's Simple 7 (continuous).

OR, odds ratio.

## Discussion

Participants with lower incomes, were younger, female, had lower Life's Simple 7 cardiovascular health scores, low BMI, and were current smokers, as well as higher rates of mild to severe depressive symptoms, which was in agreement with previous research findings (6, 37).

The results of our study indicated that SES had a linear association with ASCVD risk and cause-specific and all-cause mortality, which were consistent with those of the following studies: In developed nations such as the United States, several recent studies have indicated that the ORs for the association between CVD risk and low- and middle-incomes over the high-incomes were 1.49 (95% CI = 1.16–1.91) and 1.27 (95% CI = 1.10–1.47), respectively. Adults with lower incomes had a greater risk of death from all causes than adults with a high SES (HR = 2.13, 95% CI = 1.90–2.38) (37). People with self-reported income in the lowest income bracket between the ages of 35 and 64 were twice as likely to die from myocardial infarction and CHD as those in the highest quartile (38). There were disparities in CHD mortality between men and women, but both were nearly twice as common in the low-income group compared to the high SES group. Moreover, Odutayo et al. reported that the cardiovascular risk decreased in the high-income group from 1999 to 2014, but not in the low-income group (39).

Similar findings were observed in developing nations: as one of the middle-income nations, China has more than 17 million CVD patients (40). Total CVD prevalence was lower in high- and middle-income regions than in low-income regions (*P* = 0.0064; 7.46, 7.42, and 8.36%, respectively) (41).

The strong effect of SES cannot entirely be explained by the dependence of CVD risk factors. For example, as evidenced in the Whitehall study, men with the lowest incomes have a 10-year CHD death risk 2.7 times that of their highest incomes, which has been reduced to 2.1 after adjusting for conventional risk factors (42). This indicates that the CVD risk imparted by low SES is relatively independent of major CVD risk factors. Even at best, classical risk factors seem to be responsible for only 15–30 percent of the CVD risks associated with SES (43). It is evident that there are other unappreciated societal factors at work that could explain the relationships between ASCVD and SES.

Numerous resources (access to knowledge, wealth, power, prestige, and positive social relationships, recreational facilities), protective factors (access to healthy lifestyle and health care services), education, medical compliance, stress, nutritious food, safe communities, physical activity, smoking, alcohol consumption, drug use, and air pollution were among the potential mechanisms (43–46). For instance, extensive evidence from animal models supports the link between air pollution exposure and ASCVD, demonstrating that increased exposure to concentration ambient particles increases atherogenesis in controlled conditions (47); food insecurity was related to greater metabolic risk (48); and lifestyle factors accounted for 12.3% of the association between SES and death, according to a study (37).

Furthermore, Lower adherence to hospital visits, poorer blood pressure control, and higher risk of in-hospital mortality and post-discharge events in patients with heart failure may contribute to cardiovascular death in low-income participants (49, 50). Biological, behavioral, and psychological risk factors contribute to the occurrence of all-cause death in participants with lower incomes (51). The above explains was a host of reasons why the people with self-reported income in the low-income bracket would be at greater risk of ASCVD and cause-specific mortality and all-cause mortality.

The greatest impact of ASCVD was felt by socially disadvantaged groups. Therefore, immediate actions are required to eliminate socioeconomic health disparities and improve population resilience. Consequently, there was a need to target low-income groups with specific ASCVD management advice.

There are some limitations to the present study that deserve attention. First, the application of competitive risk model in the survival analyses was limited due to complex, stratified multistage, probability cluster design of NHANES survey. The second point to consider was whether there was any possibility that some residual and unmeasured confounders exist, which might bias the findings of our study, even though we have controlled most of the cardiovascular risk factors using weighted logistic regressions and Cox proportional hazards regressions. Third, PIR and ASCVD were all obtained from self-report, which may result in recall bias or interviewer bias. Last but not least, this was a cross-sectional study, so causality should not be claimed on the basis of these findings.

## Conclusion

Our study confirmed that the SES was strongly linked to living with ASCVD, and cause-specific and all-cause mortality, even after adjusting for other factors that could impact risk, such as the AHA's Life's Simple 7 cardiovascular health score and variables of age, sex, marital status, education, and depression severity.

## Data availability statement

The original contributions presented in the study are included in the article/Supplementary material, further inquiries can be directed to the corresponding author/s.

## Ethics statement

The studies involving human participants were reviewed and approved by the NCHS Research Ethics Review Board (ERB) for the presented surveys years: Protocol #2005–06 (NHANES 2005–2006), Continuation of Protocol #2005–06 (NHANES 2007–2010), Protocol #2011–17 (NHANES 2011–2012), Continuation of Protocol #2011–17 (NHANES 2013–2016), and Protocol #2018–01 (NHANES 2017–2018). More information about the NHANES database can be found at https://www.cdc.gov/nchs/nhanes/irba98.htm. The patients/participants provided their written informed consent to participate in this study.

## Author contributions

RS had full access to all of the data in the study and take responsibility for the integrity of the data and the accuracy of the data analysis. RS and NZ contributed equally as co-first authors. RS, NZ, and JW: concept and design. RS and NZ: drafting of the manuscript. RS, NZ, TZ, JW, PG, and SS: critical revision of the manuscript for important intellectual content. RS and SS: statistical analysis. TZ: obtained funding and supervision. TZ, DeL, and PG: administrative, technical, or material support. All authors: acquisition, analysis, or interpretation of data. All authors contributed to the article and approved the submitted version.

## Funding

This work was supported by Beijing Municipal Science & Technology Commission Program (Grant Nos. Z171100001017203 and D181100000218005).

## Conflict of interest

The authors declare that the research was conducted in the absence of any commercial or financial relationships that could be construed as a potential conflict of interest.

## Publisher's note

All claims expressed in this article are solely those of the authors and do not necessarily represent those of their affiliated organizations, or those of the publisher, the editors and the reviewers. Any product that may be evaluated in this article, or claim that may be made by its manufacturer, is not guaranteed or endorsed by the publisher.
